# Important wheat diseases in the US and their management in the 21st century

**DOI:** 10.3389/fpls.2022.1010191

**Published:** 2023-01-12

**Authors:** Jagdeep Singh, Bhavit Chhabra, Ali Raza, Seung Hwan Yang, Karansher S. Sandhu

**Affiliations:** ^1^ Department of Crop, Soil & Environmental Sciences, Auburn University, Auburn, AL, United States; ^2^ Department of Plant Science and Landscape Architecture, University of Maryland, College Park, MD, United States; ^3^ College of Agriculture, Oil Crops Research Institute, Fujian Agriculture and Forestry University, Fuzhou, China; ^4^ Department of Integrative Biotechnology, Chonnam National University, Yeosu, Republic of Korea; ^5^ Bayer Crop Sciences, Cheaterfield, MO, United States

**Keywords:** genetic mapping, rusts, climate change, sustainability, wheat

## Abstract

Wheat is a crop of historical significance, as it marks the turning point of human civilization 10,000 years ago with its domestication. Due to the rapid increase in population, wheat production needs to be increased by 50% by 2050 and this growth will be mainly based on yield increases, as there is strong competition for scarce productive arable land from other sectors. This increasing demand can be further achieved using sustainable approaches including integrated disease pest management, adaption to warmer climates, less use of water resources and increased frequency of abiotic stress tolerances. Out of 200 diseases of wheat, 50 cause economic losses and are widely distributed. Each year, about 20% of wheat is lost due to diseases. Some major wheat diseases are rusts, smut, tan spot, spot blotch, fusarium head blight, common root rot, septoria blotch, powdery mildew, blast, and several viral, nematode, and bacterial diseases. These diseases badly impact the yield and cause mortality of the plants. This review focuses on important diseases of the wheat present in the United States, with comprehensive information of causal organism, economic damage, symptoms and host range, favorable conditions, and disease management strategies. Furthermore, major genetic and breeding efforts to control and manage these diseases are discussed. A detailed description of all the QTLs, genes reported and cloned for these diseases are provided in this review. This study will be of utmost importance to wheat breeding programs throughout the world to breed for resistance under changing environmental conditions.

## 1 Introduction

**Figure 1:**
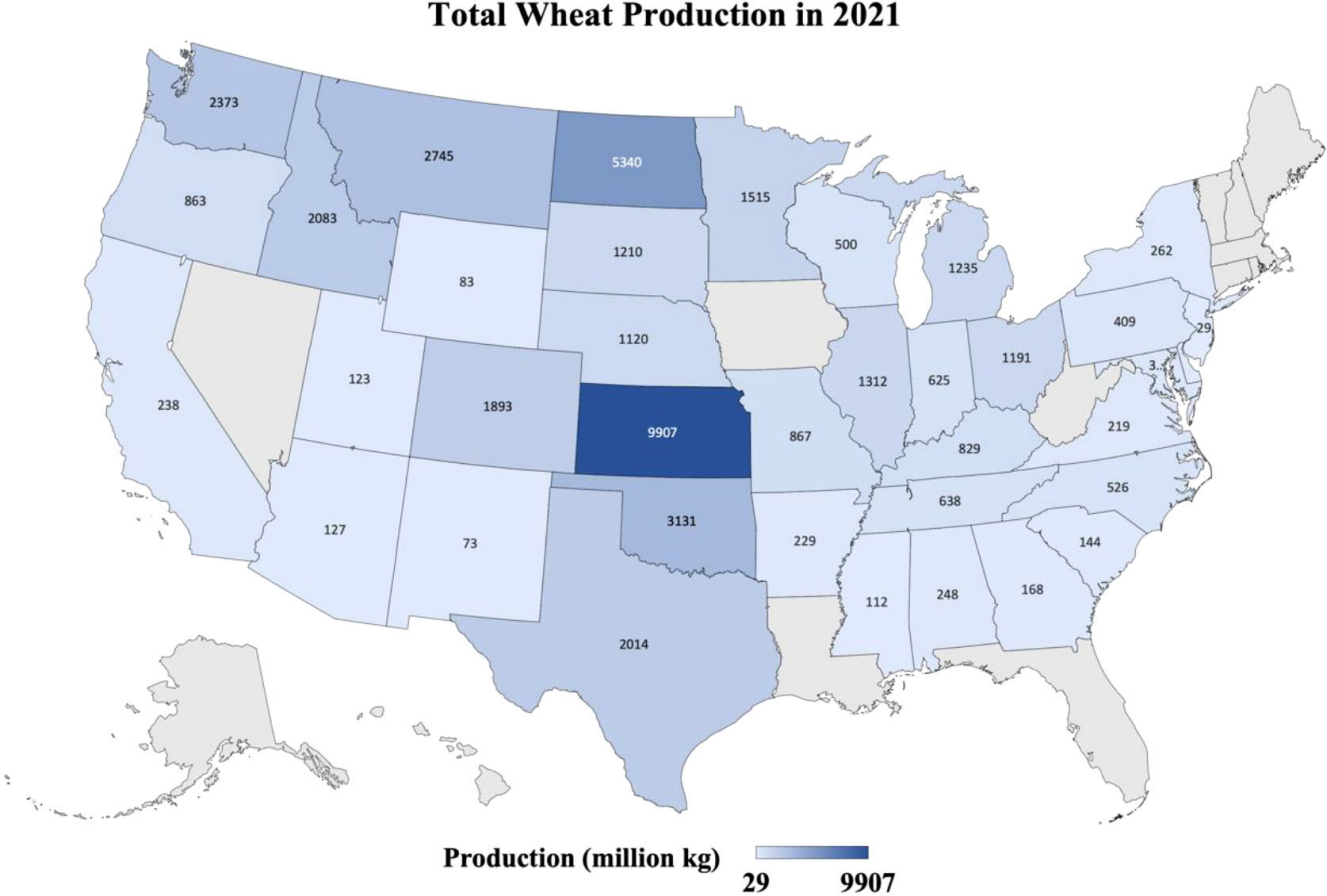
Heatmap shows the production in million kg of the wheat from different states of the USA where wheat was grown in 2021. Data retrieved from https://quickstats.nass.usda.gov/results/9A1899AA-E4AB-3E6F-B578-CD70E8143352.

Wheat is one the most important cultivated food crop across the globe. Wheat alone contributes ~205 of global human calorie intake. Annually, it is grown over an area of 219 million ha and yielding over 760 million tons. USA is the fourth largest producer and second largest exporter of wheat (www.fao.org). Wheat is attacked by over 100 different diseases caused by various pathogens and pests. Around 21.5% of wheat production is lost to these diseases annually. Globally, Leaf rust is the most economically damaging disease of wheat followed by Fusarium head blight and Tritici blotch (Savary et al., 2019) (**Figure 1**).

An integrated management strategy incorporating genetic resistance, chemical control, biological and cultural practices is needed (Simón et al., 2011). Popular management strategies employed by farmers include use of resistant varieties and fungicide application (Mesterházy et al., 2011). The first line of defense is genetic resistance which offers the most sustainable and economic strategy. Plant and pathogens are in a continuous evolutionary race with each other. Pathogens keep on evolving to new and more aggressive strains overcoming the past sources of resistance such as the case of *Ug99* stem rust (Pretorius et al., 2000; Singh et al., 2011; Kaur et al., 2021). It requires breeders and pathologists across the globe to keep looking for new sources of resistance (Momeni et al., 2014). A variety of chemicals are available to eradicated or limit the growth of pests and pathogens and is the second most important management strategy after genetic control. Fungicides also lose their effectiveness over time due to development of fungicide resistance in the pathogen population such as reduction in the effectiveness of Azole group of fungicides against *Parastagonospora nodorum* (Pereira et al., 2020). This brings into picture a notable environment friendly way for disease management by using biological control agents (BCAs). BCAs application suffers from some disadvantages including non-uniform efficacy across the fields, different environments, and limited shelf life of the BCAs (Shah et al., 2018). Cultural/agronomic practices include crop rotation, tilling, irrigation management, and manipulating planting dates. Cultural control has disadvantages like limited effectiveness and associated high costs (Wegulo et al., 2015). Therefore, an integrated management strategies is required for satisfactory disease control.

Success of breeding programs is highly dependent on the genetic variation in the breeding population, availability of the resistant germplasms, and efficient screening of the lines in fields or greenhouses for disease ratings. Traditionally plant breeders used to rely only upon field screening for the development of resistant cultivars. However, with the advent of molecular markers in the 1980s, plant breeders were the first to adopt the marker-assisted selection (MAS) for tagging the resistance locus (Bernardo, 2008; Sandhu et al., 2022a; Sandhu et al., 2022b). This further aided in the easy sharing of germplasm or gene pools across the breeding programs. High throughput genotyping technology has dramatically increased the number of molecular markers for utilization in genetic studies such as genotype by sequencing (GBS) and Illumina 90K SNP chip assay (Poland et al., 2012; Wang et al., 2014). These SNP variants allowed the rapid identification of quantitative trait loci (QTL) associated with disease resistance using linkage or association mapping. Both techniques complement each other by using the high statistical power of linkage mapping with high association mapping resolution (Yu et al., 2008; Sandhu et al., 2021b). Although several resistance genes have been cloned, still, map-based cloning is difficult in wheat due to the genome’s complexity (Rasheed and Xia, 2019; Arya et al., 2022; Gill et al., 2022).

Since the last 30 years, there have been various efforts to identify and clone the genes confirming various disease resistance (Aboukhaddour et al., 2009; Aboukhaddour et al., 2020). For example, in the case of stripe rust of wheat, 300 QTL have been identified, showing resistance to the causal pathogen. After extensive research for the QTL mapping, it has been observed that most of the QTLs have a small effect, causing a problem during introgression into another germplasm (Bernardo, 2016; Saini et al., 2021). Recently different breeding and pathologist are using genomic estimated breeding values to account for the small and large gene effects to predict the disease ratings of lines to decide parents and screening materials. (Sandhu et al., 2021c; Sandhu et al., 2021d).

This review focuses on the important diseases of wheat in the USA, their causal organism, economic damages caused. Host range, symptoms, favorable conditions, diseases management and integrated disease management. This review provides detailed information about various QTLs mapped and genes cloned for the important diseases of wheat.

## 2 Stripe rust

Stripe rust, or yellow rust, is one of the most destructive diseases in wheat, capable of causing complete crop yield loss if a susceptible cultivar gets infected at the early crop growth stage (Chen et al., 2014). More than 80% of the world’s wheat cultivars exhibit stripe rust symptoms, and approx. $1 billion loss is incurred annually worldwide (Figueroa et al., 2018). During the early 1960s, a severe stripe rust epidemic was observed in the Pacific Northwest of the United States and a loss of approx. $45 and $15 million were reported from Washington and Oregon, respectively (Line, 2002). Other major epidemics were recorded during 2003, 2005, 2010, 2012, 2015, and 2016, where disease severely infected the Great Plains, especially during later years.

Stripe rust is caused by an obligate biotrophic fungus, *Puccinia striiformis* Westend. f. sp. *tritici* (*Pst*) (Lin and Chen, 2009). F. Kolpin Ravn, a visiting scientist from Denmark, was the first to recognize the *Pst* in the USA (Carleton, 1915). Many researchers assumed that the *Pst* originated from Transcaucasia, where wild grasses served as the fungus’s primary host; from there, it spread to other parts of the world (Hovmøller et al., 2011; Chen et al., 2014). However, other researchers assumed it originated from western China and Central Asia (Mboup et al., 2009; Ali et al., 2010). However, clear information about this disease’s origin is still unavailable. Earlier, the disease was observed only in the cool temperate regions. Still, due to its wide adaptability and new aggressive strains identified after 2000, stripe rust can now be seen in the warmer temperature regions (Milus et al., 2009; Hovmøller et al., 2011).

The epidemics of stripe rust in wheat are noticed worldwide, including in parts of Australia, Asia, Africa, Europe, and North America (Wellings, 2011; Solh et al., 2012). Stripe rust can be traced over all the wheat-growing areas of the USA, but it is more prevalent and causes huge economic losses in the eastern parts of the Rocky Mountains (Chen et al., 2010). This rust is commonly found in grasses belonging to the *Aegilops*, *Agropyron*, *Bromus*, *Elymus*, *Hordeum*, *Secale*, and *Triticum* genera (Line, 2002; Hovmøller et al., 2011). In the USA, particularly the Pacific Northwest (PNW), where wheat (one of the hosts) is grown throughout the year, it enables the *Pst* spread from one season to another (Chen et al., 2014). The importance of *Berberis* spp. and *Mahonia* spp. serving as the alternative host for *Pst* has also been recently recognized (Chen et al., 2014).

### 2.1 Symptoms, favorable conditions and disease cycle

The *Pst* can infect leaves, leaf sheaths, and glumes of plants during any crop growth stage (Chen et al., 2014). The linear arrangement of pustules production on leaves after the middle jointing stage is the most important and distinguishing symptom of this rust. However, depending upon the cultivar resistance, visual disease symptoms can vary from small flecks and chlorosis in resistant cultivars to large yellow-orange colored pustules of uredia arranged in a linear fashion (stripes) in susceptible cultivars (Chen, 2005; Chen et al., 2014). Later in the crop season, these urediniospores produce telia, converting the yellow-orange spots to black-coloured teliospores (Chen et al., 2014).

Under the conducive environmental conditions of high moisture coupled with cool temperature (7–12°C), disease symptoms appear less than 10 days after the infection (Chen, 2005; Chen et al., 2014). The *Pst* fungi obtain water and nutrients by forming haustorium inside the host plant. Like other rust fungi, *Pst* produces five types of spores and completes its life cycle on two botanically distinct plants (Chen, 2020). The urediniospores having two nuclei are the repeating spores of *Pst* and cause disease infection on the primary host, mainly wheat. These urediniospores are formed during the asexual stage of the fungi and produce single nuclei teliospores afterwards on the same plant (Chen, 2020). Germinating teliospores produce basidiospores that later infect the plants of *barberry* species. The pycniospores and aeciospores are formed on the plants of *barberry* spp., and the aeciospores complete the disease cycle of the fungi by infecting the primary host (Chen, 2020). Therefore, depending upon the host, the *Pst* can survive thick-walled telia (Chen, 2020) or overwinters as mycelium or spores on the primary host (Line, 2002). The primary spread of urediniospore of stripe rust is often accomplished *via* wind (Chen et al., 2014). Raindrops also help in the dispersal of the disease over shorter distances.

### 2.2 Cultural and chemical control

The continued availability of wheat throughout the year in the United States’s PNW region makes stripe rust more frequent. Practice like crop rotation will reduce the inoculum buildup and, subsequently, the stripe rust (Chen, 2005; Carmona et al., 2020). A reduced rate of nitrogen fertilizers has also been observed to reduce the risk of stripe rust disease incidence (Devadas et al., 2014). Other cultural practices for reducing the stripe rust disease include optimum irrigation quantity and adjusting the planting date (Chen, 2007).

The first chemical control of stripe rust at a large commercial scale started during the early 1980s in the PNW (Line, 2002). Fungicides like trifloxystrobin, propiconazole, triazole, and cyproconazole are widely used to reduce the chances of fungicide resistance development in *Pst*.

### 2.3 Breeding for stripe rust resistance

Developing a resistant cultivar is the most sustainable and economical approach for reducing stripe rust’s impact on wheat production. There are mainly two types of genetic host-plant resistance against stripe rust that are mostly used to date (Carter et al., 2009; Milus et al., 2015; Chen, 2020). The first is all-stage resistance (ASR), also called seedling resistance, which typically starts expressing during the seedling stage. The ASR is race-specific, inherited qualitatively, and offers high levels of resistance against the *Pst*. However, due to its race-specific nature, it gets easily overcome by the evolving strains of *Pst*. Therefore, race-specific resistance has an advantage only over a short period. The second type of resistance is the adult-plant resistance, particularly the high-temperature adult-plant (HTAP) resistance. The HTAP resistance is expressed at later stages of plant growth under warm temperature conditions. The HTAP resistance is usually durable, non-race-specific, and inherited quantitatively. However, HTAP offers limited disease resistance against the *Pst*. Therefore, combining ASR and HTAP resistance is considered a more effective approach to control stripe rust in wheat.

Considering the impact of stripe rust on wheat production, intensive research has successfully cloned nine genes conferring resistance to *Pst*. Six of these cloned genes were identified at the B sub-genome, and the rest three were identified at the D sub-genome. Information regarding the cloned genes for stripe rust of wheat can be found in **Table 1**.

**Table 1:** List of cloned genes showing resistance to different diseases of wheat.

Disease	Pathogen	Gene	Chromosome	Cloning method	Protein encoded		References
Stripe rust	*P. striiformis* f. sp. *tritici*	*Yr5*	2BL	MutRenSeq	BED-NB-LRR	(Marchal et al., 2018)	
Stripe rust	*P. striiformis* f. sp. *tritici*	*Yr7*	2BL	MutRenSeq	BED-NB-LRR	(Marchal et al., 2018)	
Stripe rust	*P. striiformis* f. sp. *tritici*	*Yr10*	1BS	Map-based cloning	CC-NBS-LRR	(Liu et al., 2014b)	
Stripe rust	*P. striiformis* f. sp. *tritici*	*Yr15*	1BS	Map-based cloning	TKPs	(Klymiuk et al., 2018)	
Stripe rust	*P. striiformis* f. sp. *tritici*	*Yr18*	7DS	Map-based cloning	ABC transporter	(Krattinger et al., 2009)	
Stripe rust	*P. striiformis* f. sp. *tritici*	*Yr28*	4DS	Map-based cloning	NB-LRR	(Zhang et al., 2019a)	
Stripe rust	*P. striiformis* f. sp. *tritici*	*Yr36*	6BS	Map-based cloning	Kinase-START	(Fu et al., 2009)	
Stripe rust	*P. striiformis* f. sp. *tritici*	*Yr46*	4DL	Map-based cloning	Hexose transporter	(Moore et al., 2015)	
Stripe rust	*P. striiformis* f. sp. *tritici*	*YrSP*	2BL	Map-based cloning	BED-NB-LRR	(Marchal et al., 2018)	
Leaf rust	*Puccinia triticina Eriks.*	*Lr1*	5DL	Map-based cloning	CC-NBS-LRR	(Cloutier et al., 2007)	
Leaf rust	*Puccinia triticina Eriks.*	*Lr10*	1AS	Map-based cloning	CC-NBS-LRR	(Feuillet et al., 2003)	
Leaf rust	*Puccinia triticina Eriks.*	*Lr21*	1DS	Map-based cloning	CC-NBS-LRR	(Huang et al., 2003)	
Leaf rust	*Puccinia triticina Eriks.*	*Lr22a*	2DS	TACCA	CC-NBS-LRR	(Thind et al., 2017)	
Leaf rust	*Puccinia triticina Eriks.*	*Lr34*	7DS	Map-based cloning	ABC transporter	(Krattinger et al., 2009)	
Leaf rust	*Puccinia triticina Eriks.*	*Lr42*	1DS	RNA seq mapping	NLR gene	(Lin et al., 2022)	
Leaf rust	*Puccinia triticina Eriks.*	*Lr67*	4DL	Map-based cloning	Hexose transporter	(Moore et al., 2015)	
Fusarium Head Blight	*F.graminearum*	*Fhb1*	3BS	Map-based cloning and Association mapping	PFT	(Rawat et al., 2016)	
Fusarium Head Blight	*F.graminearum*	*Fhb1*	3BS	Map-based cloning and Association mapping	TaHRC	(Li et al., 2019; Su et al., 2019)	
Fusarium Head Blight	*F.graminearum*	*Fhb7*	7DL	Map-based cloning	GST	(Guo et al., 2015; Wang et al., 2020)	
Powdery Mildew	*Blumeria graminis* f. sp. *tritici*.	*Pm1*	7AL	MutRenSeq	NBS-LRR	(Hewitt et al., 2021)	
Powdery Mildew	*Blumeria graminis* f. sp. *tritici*.	*Pm8*	1BL	Homology based cloning	CC-NBS-LRR	(Singh et al., 2018)	
Powdery Mildew	*Blumeria graminis* f. sp. *tritici*.	*Pm17*	1AL	Homology based cloning	CC-NBS-LRR	(Singh et al., 2018)	
Powdery Mildew	*Blumeria graminis* f. sp. *tritici*.	*Pm21*	6AL	Micro-array based cloning	Serine/ threonine kinase	(Cao et al., 2011)	
Powdery Mildew	*Blumeria graminis* f. sp. *tritici*.	*Pm24*	1DS	Map-based cloning	Wheat Tandem Kinase	(Lu et al., 2020)	
Powdery Mildew	*Blumeria graminis* f. sp. *tritici*.	*Pm41*	3B	Map-based cloning	CC-NBS-LRR	(Li et al., 2020)	
Powdery Mildew	*Blumeria graminis* f. sp. *tritici*.	*Pm60*	7AL	Map-based cloning	NBS-LRR	(Zou et al., 2018)	
Powdery Mildew	*Blumeria graminis* f. sp. *tritici*.	*WTK4*	7DS	Associaion mapping	Wheat Tandem Kinase	(Gaurav et al., 2021)	

* TACCA, Targeted chromosome-based cloning via long-range assembly; BED-NB-LRR, BED domain nucleotide binding leucine rich repeat; NB-LRR, Nucleotide binding leucine rich repeat; CC-NBS-LRR, Coiled-coil nucleotide binding leucine rich repeat; TKPs, Tandem kinase-pseudokinases; ABC transporter, ATP binding cassette transporter; Kinase-START, Kinase steroidogenic acute regulatory protein-related lipid transfer; ABC transporter, ATP binding cassette transporter; PFT, Pore-forming Toxin; HRC, Histidine-rich calcium binding protein; GST, Glutathione-S-transferase.

## 3 Leaf rust

Leaf rust, also known as brown rust, started along with the production of wheat in the early 1700s in the USA but remained unnoticed until the early 1800s when de Candolle identified it as an individual rust disease different from the stem and other rusts (Chester, 1946). The commonly used name for the obligate fungus causing leaf rust in wheat is *Puccinia triticina Eriks.* (*Pt*) (Huerta-Espino et al., 2011). It occurs more regularly and has wide distribution compared with other cereal rusts. The conducive weather conditions for leaf rust development also favor more wheat yield response in most cultivars; thus, reduced wheat yield due to leaf rust was often ignored or underestimated (Soliman et al., 1963). However, in 1938, a significant decrease in wheat production was observed in the central Great Plains (Chester, 1946). The wheat yield’s reduction due to leaf rust results in fewer kernels and decreased 1000 kernel weight. In the USA, more than $350 million loss occurred due to leaf rust during 2000-2004 (Huerta-Espino et al., 2011). Over the last century, minor to major leaf rust epidemics have occurred with 3.7 to 50% yield damage in the Great Plains of the United States (Moschini and Pérez, 1999; Eversmeyer and Kramer, 2000). The magnitude of crop loss decreases as the crop progresses towards maturity. For instance, leaf rust infection on flag leaf occurring at the soft dough stage results in approx 8% yield damage, and the corresponding yield loss can decrease to less than 4% when infection occurs at the hard dough stage (Huerta-Espino et al., 2011).

Leaf rust is observed in all the wheat-growing areas of the world, including Asia, Eastern Europe, North America, and Australia. Although it occurs throughout the wheat-growing areas of the USA, it is of great importance in the Great Plains of the United States (Kolmer, 2005). The primary hosts are common and durum wheat, commonly found in the Middle East, Ethiopia, Europe, Mexico and South America (Bolton et al., 2008; Liu et al., 2014a). In the southern High Plains of the United States, *Pt* has been observed on the common goatgrass (*Aegilops cylindrica*). In addition, *Pt* shows occasional infection (under non-normal conditions) on limited crops like barley (Neu et al., 2003). The meadow rue (*Thalictrum speciosissimum* L.) sporadically serves as a secondary host in Portugal; however natural infection on meadow rue in the USA is rarely reported (Bolton et al., 2008; Liu et al., 2014a).

### 3.1 Symptoms, favorable conditions and disease cycle

The *Pt* is commonly found on leaves but can infect glumes and awns depending upon the disease severity. Typical symptoms include the presence of small circular to oval yellow-orange colored pustules. These orange pustules give a ‘rusty’ appearance to leaves. As wheat matures, these orange-coloured urediniospores develop into brown-black spores known as teliospores (Bolton et al., 2008). The *Pt* is classified under cold temperature species and requires 16°C as the optimum temperature for completing the infection process (Eversmeyer, 1988). Leaf rust develops under favorable environmental conditions of high humidity with a 10-30°C temperature range (El-Orabey and Elkot, 2020). The symptoms can be seen a week or two after the infection. *Pt* is a heteroecious macrocyclic fungus that requires two different hosts, where wheat serves as the principal host and meadow rue (*Thalictrum speciosissimum* L.) as the alternative host. To complete the life cycle, *Pt* produces five different spores, of which three of them, viz. urediniospores, teliospores and basidiospores, occur on cereal hosts, and the other two, viz. pycniospores and aeciospores, occur on the alternative hosts (Bolton et al., 2008; Song et al., 2011; Kolmer, 2013). The dikaryotic, globoid-shaped urediniospores infect the top surface of leaves and start the primary infection. These urediniospores have the potential to cycle continuously on the primary hosts.

Later during the infection, when the host plant matures, these urediniospores produce diploid nuclei containing teliospores. Further, these teliospores produce basidiospores that infect the alternate host, ultimately forming pycniospores that produce the dikaryotic nuclear condition. The dikaryotic mycelium develops into aecium containing dikaryotic aeciospores. Once the aecium gets matured, it releases aeciospores that get disseminated by the wind. The asexual urediniospores are produced after the successful establishment of aeciospores on the primary host, and thereby *Puccinia triticina* completes its disease life cycle (Eversmeyer and Kramer, 2000; Bolton et al., 2008; Kolmer et al., 2009; Kolmer, 2013; Figueroa et al., 2018). In the USA, the alternative host of *Puccinia triticina* does not occur naturally, which renders the fungus infection as urediniospores only. The *Puccinia triticina* survives as mycelium during winter or as urediniospores on host plants in the southern Great Plains of the United States (Kolmer, 2013).

### 3.2 Cultural and chemical control

Removal of volunteer wheat, planting early maturing cultivars, and applying nitrogen fertilizers at recommended doses can prove helpful in managing leaf rust (Boquet and Johnson, 1987; Moschini and Pérez, 1999; Eversmeyer and Kramer, 2000). In addition, azoxystrobin, strobilurin, and picoxystrobin-based fungicides can be used to control the disease epidemic; however, their application is discouraged due to reduced economic benefits (Hak et al., 1980; Nagarajan and Joshi, 1985; McCallum et al., 2016).

### 3.3 Breeding for leaf rust resistance

Studies on leaf resistance started back in the 1900s, when researchers found *Lr1* and *Lr2* genes conferring resistance against leaf rust in cultivar “Malakof” and “Webster”, respectively (Mains et al., 1926). More than 249 QTLs have been identified at different chromosome regions exhibiting leaf rust resistance in wheat (Mu et al., 2020). Two host-plant resistance mechanisms have been found for wheat leaf rust disease (Bolton et al., 2008; McCallum et al., 2016) The first one is race-specific resistance which shows a hypersensitive response (HR) to the pathogen but may not show the same resistance to all the pathogen isolates. Due to race-specific resistance and short time control against *Pt*, they are known for their ‘boom-and-bust cycles’. They carry matching avirulence genes and encode proteins with nucleotide binding site-leucine rich repeat (NBS-LRR) (Bolton et al., 2008; Hulbert and Pumphrey, 2014). The second type is non-specific resistance, also called adult plant resistance (APR). The APR is quantitative resistance in nature and shows equal resistance to all pathogen isolates (German and Kolmer, 1992; Periyannan et al., 2017). The virulent strains of leaf rust are evolving at high speed. This is mainly due to the presence of race-specific resistance in most of the cultivars. So, it is suggested to incorporate non-specific resistance genes like *Lr27*, *Lr34*, *Lr46*, *Lr67*, and *Lr68* into new cultivars to have more durable and steady resistance against leaf rust (Ellis et al., 2014). However, planting the same cultivar with specific or nonspecific resistance over a large area is highly discouraged to avoid further evolution of the pathogen strain. **Table 2** highlights the detailed information on the cloned genes, their chromosomal locations and protein encoded for conferring leaf rust resistance in wheat.

## 4 Fusarium head blight

Fusarium head blight (FHB), also known as head scab, is one of the most destructive diseases of grain crops as it has adverse impact on both quantity (yield losses) and quality (toxin production). It is a disease of the humid and sub-humid environment of the temperate and subtropical regions (Bai and Shaner, 1994). The FHB was first described by W.G. Smith in 1884 in England as “Wheat Scab”. In the USA, FHB was documented as a disease affecting wheat production in Indiana and Ohio, respectively in the early 1890s. FHB is caused by fungi of *Fusarium* species, a hemi-biotrophic, filamentous ascomycete. *Fusarium graminearum (F. graminearum*), *F. avenaceum* and *F. culmorum* are the major species reported to cause FHB (Parry et al., 1995). However, *F. graminearum* is the most predominant in most regions of the world (Parry et al., 1995; Goswami and Kistler, 2004; Xu and Nicholson, 2009; McMullen et al., 2012).

*F. graminearum* deteoraites crop quality by producing trichothecene mycotoxins: Deoxynivalenol (DON, vomitoxin), acetyl deoxynivalenol and nivalenol amongst which DON is the most predominant one (Chen et al., 2019). DON is phytotoxic for plants and can cause wilting, chlorosis, and necrosis. It inhibits protein synthesis in mammals by binding to 60S subunit of eukaryotic ribosomes (Rocha et al., 2005). In wheat, DON acts as a virulence factor for *Fusarium*, helping the pathogen in disease spread (Jansen et al., 2005). Food and Drug Administration has set an advisory limit of 1 ppm DON for wheat and barley products for consumption, 10 ppm for ruminating cattle and chicken, 5 ppm for swine and all other animals.

FHB outbreaks caused an estimated loss of $1 billion in 1993 in tri-state areas of Minnesota, North Dakota and South Dakota and Manitoba (McMullen et al., 1997). From 1993-2001, the northern Great Plains and central United States suffered a total loss of $7.7 billion (Nganje et al., 2004). Then, in 2003, a regional epidemic occurred in south-eastern USA, resulting in a total loss of $13.6 million (McMullen et al., 2012). During 2015-2016, economic losses by FHB damage in the USA have been estimated to be $1.2 billion (Wilson et al., 2018).

The pathogen attacks a number of cereals: wheat, barley, oats, rice, maize and sorghum (Parry et al., 1995). This pathogen was also isolated from different non-graminaceous weed species belonging to families: Solanaceae and Asteraceae which could serve as alternate hosts for this pathogen (Mourelos et al., 2014).

### 4.1 Symptoms, favorable conditions and disease cycle

Initial symptoms of FHB are best described as small, water-soaked brown colored spots at the base or middle of glume or on rachis, which later on spread in all directions on the spikes. Premature bleaching of spikelets is one the characteristic symptoms of FHB. Later on, orange coloration can be seen in infected spikelets due to presence of conidia and white mycelial growth can be observed on glumes. During prolonged warm and moist weather conditions, blue or black coloration can be seen over spikelets giving “scabby” appearance. Brown to black discolorations can also be seen over rachis and culm at later stages of disease. Infected florets will either be sterile or produce shriveled “tombstone” kernels with chalky/floury appearance. The infected light-weight seeds will have poor germination rates and poor crop stand (Bai and Shaner, 1994; Parry et al., 1995).

Wheat is susceptible to FHB from the anthesis stage up to the soft dough stage (McMullen et al., 2012). After successful colonization, the fungus starts producing mycotoxins which are translocated to other parts of the plant *via* xylem and phloem (Brown et al., 2010).

Temperature and moisture have a very critical role in the occurrence of FHB (Xu and Nicholson, 2009). A warm, moist environment with temperatures varying from 25-30°C characterized by frequent precipitation or heavy dew at the time of anthesis is highly favorable for fungal growth, infection, disease development and spread. The pathogen can overwinter as perithecia on mature cereal crops, crop debris or on infected seeds. Fungus can also overwinter as mycelia or chlamydospores (Guenther and Trail, 2005; Trail, 2009). Perithecia can be viable for up to 16 months on maize kernels and up to 23 months on wheat straw residue. Airborne ascospores serve as the primary inoculum of the disease. The rain-splashed or wind-blown conidia from infected tissues is the secondary source of inoculum to flowering heads (Guenther and Trail, 2005; Trail, 2009).

### 4.2 Cultural, chemical and biological control

Rotating wheat and barley with non-host crops such as alfalfa and soybean reduce inoculum load. Burying infested crop residue into soil by tillage operations also helps in decreasing inoculum (Osborne and Stein, 2007). Early planting may also help in disease escape (Choo et al., 2014). Preventing excess moisture in the field at times of high disease susceptibility results in less FHB development and lower DON content (Cowger et al., 2009). Closed flowering cultivars and tall varieties were found to be more resistant to FHB (Mesterházy, 1995; Yoshida et al., 2007).

Fungicide application integrated with good disease forecasting models such as FHB prediction center and risk assessment tool (http://www.wheatscab.psu.edu) are useful tools for FHB control. Fungicide Resistance Action Committee (FRAC) code 3 fungicides, which are Demethylation inhibitors, as the most effective fungicides against FHB (Paul et al., 2018). To maximize the control efficacy, these fungicides need to be applied at the time of flowering leaving a narrow window from anthesis stage to six days after anthesis. The problem persisting with chemical control is that time of the fungicide application coincides with rain reducing the efficacy of the fungicide (McMullen et al., 2012; Shah et al., 2018).

Some bacteria (*Bacillus* spp. (Palazzini et al., 2016; Chen et al., 2018), *Pseudomonas* spp. (Schisler et al., 2006) and fungi (*Trichoderma* spp., *Cryptococcus* spp., *Aureobasidium pullulans* (Wachowska and Głowacka, 2014) were found to have adverse effects on *F. graminearum*. The mode of action includes competition for nutrients, induction of localized resistance and antagonism (McMullen et al., 2012; Wegulo et al., 2015; Shah et al., 2018). Even some of the wheat endophytes like *Sarrocladium kiliense* and *Phoma glomerata* were found to have antagonistic effects on Fusarium (Comby et al., 2017). It was found that application of BCAs is most effective when used in integration with fungicides (McMullen et al., 2012; Wegulo et al., 2015; Shah et al., 2018). Plants also produce certain metabolites like phenolic compounds and oils which provoke inhibitory responses against some important target proteins of *F. graminearum* (Cuperlovic-Culf et al., 2016).

### 4.3 Breeding for fusarium head blight resistance

Genetic resistance against FHB is complex, quantitatively controlled, and greatly affected by environmental factors like inoculation methods, amount of inoculum used, relative humidity, temperature, phenology and plant health (Ma et al., 2020). It can be classified into five types: Type-1 (resistance against initial pathogen infection) (Schroeder and Christensen, 1963), Type-2 (resistance against pathogen spread in the spike) (Schroeder and Christensen, 1963), Type-3 (resistance against toxin accumulation or ability to detoxify toxins released) (Miller and Arnison, 1986), Type-4 (resistance towards kernel infection) (Mesterházy, 1995; Mesterházy et al., 1999) and Type-5 (tolerance) (Mesterházy, 1995; Mesterházy et al., 1999). All types of resistance are generally correlated. Type-1 and Type-2 are widely studied types of resistance mechanisms. Type-3 resistance has gained attention in recent years because of the quality aspects (Brar et al., 2019).

Variation in resistance to FHB exists in primary, secondary as well as tertiary gene pool (Buerstmayr et al., 2020; Ma et al., 2020). Nearly 500 QTLs covering the whole genome of wheat have been reported in the literature (Buerstmayr et al., 2020). However, there are only seven QTLs (Fhb1 to Fhb7) which are designated as behaving as mendelized genes. These seven mendelized QTLs were derived from hexaploid wheat and provide either Type- I or Type- II resistance. *Fhb1* is the most extensively studied and widely used and explains 60% of total phenotypic variation (Bai and Shaner, 2004). Cloned genes with FHB are compiled in **Table 2**. Durum wheat is more susceptible than bread wheat (Steiner et al., 2019). Wild tetraploid wheat: *T. dicoccoides* (Buerstmayr et al., 2012) and *T. carthlicum* (Sari et al., 2018) were also found containing FHB resistance QTLs. In durum, resistance QTLs were found associated with morphological traits: *Rht-B1* gene on 4B controlling plant height (Buerstmayr et al., 2012) or q (spelt wheat genotype) allele (Zhang et al., 2014). Due to the quantitative nature of resistance provided by these QTLs, achievement of completely resistant cultivars is difficult. To develop a high level of overall resistance to FHB, multiple QTLs effective in diverse genetic backgrounds are required. For Type-3 resistance, wheat *UGT* gene *TaUGT4* (Ma et al., 2015) and two ABC transporters *TaABCC3.1* and *TaABCC3.2* (Walter et al., 2015) were reported to detoxify DON.

Engineering susceptibility genes were also found to be imparting resistance against FHB. Wheat RPL3 gene family, *Ethylene Insensitive* 2 (EIN2), *TaLpx-1* gene and transcription factor gene *TaNFXL1* were reported as susceptibility genes for FHB in wheat (Lucyshyn et al., 2007; Brauer et al., 2019). EMS mutagenized populations can be utilized to find resistance against FHB (Rawat et al., 2019; Singh et al., 2019; Chhabra et al., 2021a). Chromosome arms 2DS, 3DL, 4DS and 7AS were also reported in the literature to be harboring major effect susceptibility genes for FHB (Basnet et al., 2012; Garvin et al., 2015; Hales et al., 2020; Chhabra et al., 2021b).

## 5 Snow mold

Snow mold diseases of winter wheat occur mostly in the PNW region of the United States. These diseases occur in the field where soil remains frozen or snow cover persists for more than 100 days (Bruehl and Cunfer, 1971). Although the snow cover helps in protecting the wheat from freeze damage but also creates a favorable environment for pathogen growth (Bruehl and Cunfer, 1971). It was first observed in the USA in 1923 from southern Idaho and from 1923 to 1930, the incidences of these diseases started appearing in the states of Washington and Montana (Murray et al., 2000).

Currently, in the USA, four snow mold diseases are present, which are caused by seven different fungal pathogen strains. These diseases include speckled snow mold, pink snow mold, snow scald, and snow rot. Speckled snow mold is caused by either *Tphula idahoensis*, *T*. *ishikariensis*, or *T. incarnata*. Similarly, pink snow mold, snow scald, and snow rot are caused by *Fusarium nivale*, *Myriosclerotinia borealis* and *Pythium iwayami*, respectively. Speckled snow mold is more destructive than widely prevalent pink snow mold, while snow scald and snow rot have minimal impact and distribution in the PNW (Murray et al., 2000; Nolin and Daly, 2006).

These diseases are observed in eastern Japan, western USA and Canada, parts of Russia, central and eastern Europe. In the USA, these diseases are more prevalent in the states of Washington, Idaho, Montana, Oregon, and Utah (Bruehl and Cunfer, 1971). The accurate damage estimate from snow mold does not exist because of the sporadic infection. During significant infection years, it requires the reseeding of the crop in the spring. It is recommended that the decision for replantation is performed after two to three weeks of snow melting. It is suggested that if there are more than eight plants per square foot on average, replantation is not going to increase the profit (Murray et al., 2000). The last prolonged snow cover occurred during the winter of 2016-17 and caused significant damage to the crop in the PNW (Lozada et al., 2019). The host range of these diseases ranges from lawns, wild grasses, winter barley and wheat.

### 5.1 Symptoms, favorable conditions and disease cycle

Snow mold diseases are caused by cold-loving soil-borne fungi classified as psychrophiles. In the case of speckled snow mold, symptoms appear as whitish-gray fungal mycelial growth, which is matted to the soil. After a few days of dry and sunny weather, fungal growth disappears, leaving visible dark colored bodies called sclerotia on the infected plant parts i.e. leaves and stem. The sclerotia of T. ishikariensis and T. idahoensis are round with dark brown to black color while T. incarnata has irregular shaped reddish-brown sclerotia that are mostly present on roots. The symptoms for pink snow mold include white fungal mycelial growth on the leaves, which subsequently changes to salmon color, resulting in the name pink snow mold. In contrast to speckled snow mold, leaves in pink snow mold remain intact as opposed to speckled snow mold where the disintegration of leaves occurs (Murray et al., 2000; Iriki et al., 2001).

Snow mold growth is favored by the autumn rain and snow fallen on unfrozen soil, which persists for more than 100 days. Deep snow cover increases the chances of disease spread by raising the contact between soil and leaves (Matsumoto, 2009). Furthermore, photosynthesis stops under deep snow cover, which results in depletion of carbohydrates, making plants more susceptible to the infection (Sugiyama and Shimazaki, 2007).

The pathogen causing snow mold diseases survives as sclerotia in the soil and infected residues (Murray et al., 2000). Speckled snow mold occurs with the germination of sclerotia under snow cover, and infection begins within one month. The infection of pink snow mold occurs from residues of previous year’s infected plants. Fungal hyphal filaments from the infected residues penetrate the leaves, and infection continues to grow as long as snow persists. Pink snow mold produces airborne conidiospores which act as secondary inoculum during the spring and summer, but they are not important for disease development at that time period (Murray et al., 2000; Kruse et al., 2019).

### 5.2 Cultural and chemical control

Snow mold diseases could be reduced by decreasing the accumulation of sclerotia in the soils by removing the diseased plant residues. Rotation of winter wheat with spring wheat or legumes will slow down the rate of fungal growth and will help in the decomposition of previous soil-borne inoculum (Murray et al., 2000). Furthermore, early sown wheat varieties in august have better tolerance to snow mold because plants have enough tillers and biomass, and ultimately better spring growth (Nishio et al., 2008). Late sown wheat varieties also show tolerance to snow mold because they escape the disease entirely. Late planting is usually avoided because it delays the maturity of the crop in addition to the reduction in wheat yield (Bruehl and Cunfer, 1971). Snow blackeners such as fly ash, coal dust, or lamp black are applied over the crops to hasten the melting rate of snow (Murray et al., 2000). Fungicides containing mercury as an active ingredient are used to control snow mold when applied early in fall. The use of fungicides is avoided because of the unpredictability of the disease occurrence.

### 5.3 Breeding for snow mold resistance

Genetic diversity of resistance to snow mold is very less in the cultivated hexaploid wheat. Over 15,000 cultivars were screened in the PNW region and only ten cultivars that were showing resistance to the snow mold were identified (Bruehl, 1982). In a screening of 481 accessions of Triticum and Aegilops, only Aegilops cylindrica accessions were found resistant to speckled snow mold (Iriki et al., 2001). Breeding for snow mold is difficult due to varying amounts of snow per year, and screening is difficult because snow cover is highly variable within the same field (Bruehl, 1982). QTLs associated with the snow mold are compiled in **Table 2**.

## 6 Eyespot

Eyespot, also known as straw breaker foot rot of wheat, is an important disease prevalent in areas where wheat is grown continuously under moist and cool conditions (Murray, 2010). The name straw breaker originated as infected plants have broken stems, which ultimately result in lodging of the plants (Lucas et al., 2000). The disease is more prevalent on fall sown wheat but occasionally observed on early planted spring wheat, barley, rye, and oats (Murray, 2010). Eyespots are caused by two necrotrophic soil-borne fungal pathogens, *Oculimacula acuformis*, and *O*. *yallundae* (Crous et al., 2003). These pathogens can co-exist in the field, causing almost identical symptoms and follow the same disease cycle.

Eyespots are reported in most wheat growing countries, but it is more prevalent in the temperate climate of Pacific Northwest USA, New Zealand, and western Europe (Zanke et al., 2017). In the USA, this disease is frequently observed in Oregon, Idaho, Washington, Montana, Utah, Kansas, North Dakota, and Oklahoma (Murray, 2010). The accurate damage from eyespot does not exist because of the sporadic infection. However, under severe disease pressure, yield losses of up to 50% have been reported in farmers’ fields (Mcvay et al., 2010). The host range varied from winter wheat, spring wheat, barley, rye, and oats.

### 6.1 Symptoms, favorable conditions and disease cycle

Initially, dark lens-shaped lesions are observed on the coleoptile and outer leaf sheath near the ground during autumn or early spring. Such lesions grow deeper and darker until the jointing and heading (Li et al., 2005; Mcvay et al., 2010). The later infection spreads inwards from sheath to the stem, reducing the transportation of the nutrients and water, which weakens the stem and results in lodging. Lesions on the stem have eye-shaped or elliptical lesions with a dark brown perimeter (Cadle et al., 1997; Burt et al., 2010). Infected tillers mature early and develop white ears with no filled grain, and these tillers may fall, if infected severely. Lodged stems break off when pulled, and lodging is typically observed in multiple directions instead of one direction by rain or wind.

Disease is favored by dense canopy, high moisture, high soil nitrogen content, early seeding and continuous winter cereal crop cultivation (Vera and Murray, 2016). Under these favorable conditions, fungi produce millions of conidiospores on infected debris. Both causal organisms differ regarding isozymes, sensitivity to fungicides, pathogenicity, cultural characteristics, stem breaking strength, and ribosomal internal transcribed spacer sequence (Crous et al., 2003). Infection initiates from the mycelium or conidia present on the previous year debris or stubbles from the infected crop in the field, which are spread over the distance with rain and moisture (Vera and Murray, 2016). The most conducive conditions for spore germination and disease dispersal include a temperature in the range of 7-13°C and high humidity (Mcvay et al., 2010). When infection occurs early in the autumn, extensive colonization occurs around the stem, interfering with water and nutrient flow to the grains, resulting in shriveled grains, lodging, and white-heads (Burrows, 2013).

### 6.2 Cultural and chemical control

The disease development could be avoided by late sowing, rotation with non-host crops like canola, peas and beans to allow host debris to decay, and cultivation of spring wheat to avoid favorable conditions for pathogen growth (Burrows, 2013). Furthermore, lower seeding density is recommended for increasing the canopy airflow to reduce the disease progress (Burrows, 2013). Fertilizer application indirectly favors pathogen spread by increasing plant growth.

In regions where eyespot susceptible varieties are grown, fungicide(s) application is encouraged before the stem elongation. After jointing, the application of fungicides is of no benefit (Hoare et al., 1986). The application of fungicides could be decided by randomly sampling ten plants from the field, and if 10% of the tillers have eyespot lesions, the application of fungicides is justified (Jones, 1994). Due to extensive utilization of group 1 fungicides, namely carbendazim, benomyl, and thiophanate, resistance is developed in pathogens, and they are no longer useful, but farmers are still using them in some regions of the USA (Burrows, 2013).

### 6.3 Breeding for eyespot resistance

In the USA, there are two known genes mapped for resistance to the eyespot, namely, Pch1 and Pch2 genes. Pch1 was introduced into the wheat line VPM-1, from the Aegilops ventricosa as a single dominant gene, and was mapped on the 7D chromosome (Worland et al., 1988). The second gene, Pch2 mapped on chromosome 7A, acted as a partially dominant gene, and was introduced from the French cultivar “Cappelle Desprez” Pch2 is observed to be less resistant to the O. yallundae (Burt et al., 2010). These genes provided resistance to some extent, but a search for the additional gene is in progress to broaden the resistance’s genetic base. Several studies have identified the genes and QTLs from the wild relatives of wheat conferring resistance to eyespot, namely, T. monococcum, T. tauschii, A. longissimi, and Dasypyrum villosum (Cadle et al., 1997; Yildirim et al., 2000; Li et al., 2005). As introgression of genes from wild relatives is problematic in the breeding program due to associated linkage drag and adaptation problems, genes presented in adapted germplasm are preferred (Sandhu et al., 2021a). Hundreds to thousands of lines were screened for variability to resistance to the eyespot (Börner et al., 2006). It was concluded that some other genes are present in the present-day hexaploid wheat for providing durable resistance. Recently, studies have identified several QTLs conferring resistance to eyespots in different regions of the world in addition to the two major genes identified previously (Burt et al., 2010; Lewien et al., 2018). QTLs associated with eyespot are compiled in **Table 1**.

## 7 Cephalosporium stripe

Cephalosporium stripe is a vascular disease of winter wheat and associated cereals, caused by a soil-borne pathogen named *Cephalosporium gramineum* (Murray, 2006). Symptoms appear in the spring when winter wheat starts regrowing. Spring wheat escapes the infection, as it is planted after the infection period. This disease was first reported in Japan in 1933, and the first incidence of its occurrence in the WA, USA was in 1955 (Bruehl, 1957).

This disease is more prevalent in winter wheat growing states of northwest and midwest, namely, Nebraska, Washington, Oregon, Kansas, Idaho, Montana, and North Dakota (Bockus et al., 1994; Froese et al., 2016). Yield losses vary from 0 to 100% depending upon disease severity. The losses vary from premature tillers’ death to reduced seed sets (Quincke et al., 2014). Under extreme disease conditions, it causes significant losses to farmers by reducing grain yield resulting from reduced seed weight and number of seeds per plant (Mcvay et al., 2010; Burrows, 2013). The host range includes winter cereals such as wheat, barley, rye and oats.

### 7.1 Symptoms, favorable conditions and disease cycle

Two to three yellow stripes per leaf are the characteristic symptoms of cephalosporium stripe. As the disease progresses, yellow streaks become necrotic due to colonization of xylem cells with the fungus (T.D. Specht and Murray, 1989). The stripes become less evident over the sheath with time; however, stripes eventually appear on the flag leaves. Infection leads to stunted plants, giving the appearance of a double canopy of healthy and infected plants (Quincke et al., 2011; Vazquez et al., 2015).

Fungus growth is favored by wet soils, root injury, 7-13°C, continuous small grains cultivation, and low pH soils (< 6) (Murray, 2006). Pathogen survives as mycelium or conidia on residues on or near soil surface. The pathogen can survive on residues for three years but cannot survive in soil for more than a couple of months. Under favorable conditions, the fungus produces conidiospores on plant debris inside the ground, which is washed into layers of soil near the crown of the health crop to initiate the infection (Smiley et al., 2009). The entry of pathogens in plants is facilitated by root injury due to frost damage, mechanical damage, or root-feeding insects. Once inside the roots, fungus can colonize the entire plant (Quincke et al., 2014).

### 7.2 Cultural control

Crop rotation with a non-host crop such as maize or soybean helps reduce the potential of infection (Murray, 2006). Late sowing, deep plowing, lime addition, and the burning of crop residues have shown the potential for managing this disease, and no chemicals are currently registered for its control (Stiles and Murray, 1996; Burrows, 2013).

### 7.3 Breeding for cephalosporium stripe resistance

Currently, completely resistant cultivars are not available, and hence cultivation of moderately resistant cultivars are encouraged in the PNW (Carter et al., 2013; Carter et al., 2014; Carter et al., 2020). QTLs associated with cephalosporium stripe are compiled in **Table 1**.

## 8 Common root rot

Common root rot of wheat is a soil-borne disease. The causal agent is a saprophyte fungus *Bipolaris sorokiniana* (sexual stage of *Cochliobolus sativus*) (Kumar et al., 2002). Compared with other diseases, including fusarium, crown, and foot rot, chances of common root rot occurrence are sparse. The yield loss due to common root rot is mainly due to reduced plant emergence (Hill, 1983), reduction in the number of heads per plant and reduction in the kernel weight (Ledingham et al., 1973), although the amount of crop yield loss depends on the resistance/tolerance of the cultivar. For example, a yield loss of 13.9-23.9% was observed in susceptible cultivars and 6.8-13.6% in moderately resistant cultivars (Wildermuth et al., 1992).

The disease can be seen in any part of the world, growing small grains, mainly wheat and barley. However, the level of disease development can vary from region to region (Mathre et al., 2003). It is of great importance, particularly in the Great Plains of the United States (Tobias et al., 2009). *B. sorokiniana* infects wheat and barley, the most important hosts for their global impact on the food production basket (Kumar et al., 2002).

### 8.1 Symptoms, favorable conditions and disease cycle

The most distinguishing symptoms of common root rot include dark brown lesions on the coleoptile. The symptoms can be seen in the subcrown internode region. First, several small oval-shaped necrotic lesions can develop on the seedlings’ subcrown region. Later, as the plant develops, these multiple lesions coalesce and cover the entire subcrown internode region below the soil level resulting in a weak root growth system. Infected seedlings become stunted and show reduced tillering (Ledingham et al., 1973; Mathre et al., 2003). Warm weather conditions having soil temperature in the range of 16-40°C induce disease development and makes plants more susceptible to infection and colonized by soil-borne inoculum already present in the soil.

The *Bipolaris sorokiniana* fungus survives through thick-walled conidia and as mycelium in soil or crop debris. The *Cochliobolus sativus* (sexual stage) produces ascocarps; however, it does not contribute significantly to the disease cycle. The primary inoculum starts with mycelium-infected seed and conidia present in the soil. Once conducive conditions are met, conidia start germinating on the susceptible hosts and start the primary infection. *B. sorokiniana* penetrates the host plants *via* epidermis and wounds. Appressoria and infection pegs help the pathogen extend the infection from the epidermis to the endodermis and lead to tissue disruption, and later infected tissues disintegrate. Infected plants get colonized as the conidia spread increases. Secondary infection of the disease can happen through soil, wind and water splashes. The spread of conidia (secondary inoculum) does not support continued disease progression; however, it can serve as an inoculum for the succeeding crop season (Acharya et al., 2011).

### 8.2 Cultural, biological and chemical control

To avoid warm temperature conditions, wheat planting should be done early in spring or fall, and planting needs to be adjusted so that the top-soil temperature is no greater than 13°C. Therefore, identifying cultivars with more tolerance to reduced temperature can help increase the total production (Singh et al., 2021). Excessive application of nitrogen fertilizer has been recorded to increase disease incidences. Rotating the field with broadleaf crops like safflower and mung bean helps break the disease cycle (Wildermuth and McNamara, 1991; Tobias et al., 2009). In a greenhouse study, successful control for common root rot using a bacterial strain *Lysobacter enzymogenes* C3 was also recorded (Eken and Yuen, 2014). Seed treatment with recommended systemic fungicides such as Captan, Mancozeb, and Maneb also helps control the disease (Acharya et al., 2011).

### 8.3 Breeding for common root rot resistance

Most available wheat cultivars show low-moderate disease resistance against common root rot. The quantitatively inherited resistance for common root rot also makes it difficult to develop resistant cultivars using traditional breeding approaches. Therefore, identifying/developing cultivars exhibiting high resistance in wheat offers vast opportunities (Conner et al., 1989; Dong et al., 2010). A list of QTLs associated with common root rot is compiled in **Table 1**.

## 9 Bunt diseases

Bunt diseases adversely affect attributes of grain quality (Muellner et al., 2020). The important bunt diseases of wheat observed in the USA are dwarf, common, and Karnal bunt. Common and dwarf bunt are commonly known as stinking smut, while Karnal bunt is called partial bunt (Chen et al., 2016). Stinking bunt got its name because of the fishy odor produced by the spores inside the bunt balls. Karnal bunt is known as partial bunt as only part of grain gets infected. Common bunt is caused by *Tilletia caries*, and *T*. *foetida*, dwarf bunt by *T*. *controversa*, and partial bunt by *T*. *indica*. Karnal bunt was first reported in India in 1930, and it appeared for the first time in the USA in Arizona in 1996 (Mitra, 1931; Ykema, 1996).

These diseases are reported in India, Mexico, Nepal, Pakistan, Brazil, Iran, South Africa and the United States. These diseases are more prominent in Arizona, California, Texas, New Mexico, Idaho, Nebraska, Kansas, and Oregon, and declared the pests for the quarantine importance (Ykema, 1996; Chen et al., 2016). In the USA, an initiative to eradicate Karnal bunt started in 1996, which now restricted this disease to Arizona only. Yield losses from bunt diseases are generally less; losses up to 40% are reported for the highly susceptible cultivars (Chen et al., 2016). Grains containing more than 3% of infected kernels are avoided for human consumption. The most significant impact of these diseases has been observed on wheat export from the USA to east Asia (Chen et al., 2016). Bunt diseases can infect wheat, triticale, rye, and other related grasses, but not barley.

### 9.1 Symptoms, favorable conditions and disease cycle

Infection for Karnal bunt occurs in few spikelets per head and is not detected until maturity. For diagnosing the disease, the grain must be threshed. The infected kernels are dark and emit the characteristic fishy odor due to the presence of trimethylamine toxin (Mujeeb-Kazi et al., 2006). Similarly, stinking smut results in the replacement of kernels with brown-black spores called bunt balls which contain the fungus’s teliospores (Castlebury et al., 2005).

Bunt diseases are favored by temperature in the range of 16-21°C, and humidity greater than 80%, at heading or flowering stages (Wang et al., 2019). These diseases produce three types of spores, namely, teliospores, primary sporidia, and secondary sporidia (Wang et al., 2019). All these spores could be windborne or splashed by rain to initiate the infection. Primary source of infection is teliospores found on soil and crop residues, and their germination produces the primary sporidia (Muellner et al., 2020). Germination and multiplication from primary sporidia give rise to secondary sporidia that infect the wheat glumes. Mycelium spread from the glumes to infect the kernels (Carris et al., 2006).

### 9.2 Cultural and chemical control

Controlling irrigation at booting or flowering stage, deep plowing, and planting of cover crops helps in managing the multiplication of the initial disease inoculum. Seed treatment with fungicide triazole (Demethylation inhibitors, group 3) effectively manages the bunt diseases, particularly if every kernel is completely covered, it is recommended because of lack of resistant cultivars (Samantara et al., 2021).

### 9.3 Breeding for bunt resistance

Till now, there is no complete resistance to any of these bunt diseases in the US. Several studies have mapped the loci controlling the resistance and will be used in marker assisted selection for releasing resistance cultivars in the future. QTLs associated with bunt diseases are compiled in **Table 1**.

## 10 Fusarium crown rot

Fusarium crown rot (FCR), also known as dryland root rot or foot rot, is one of wheat’s soil-borne diseases caused by several *Fusarium* species. However, *Fusarium pseudograminearum* and *Fusarium culmorum* are the most commonly observed species causing this disease (Paulitz, 2006; Jin et al., 2020; Özer et al., 2020). Surveys conducted in the wheat-growing regions of the United States have indicated that *F*. *pseudograminearum* is more commonly observed at higher temperatures with drier conditions than *F*. *culmorum* (Kazan and Gardiner, 2018). Globally it can be found in Australia, North and South America, Parts of Africa, Asia, and Europe. In the PNW of the United States, this disease has caused an average yield loss of 9% with a maximum of 35% (Poole et al., 2012).

### 10.1 Symptoms, favorable conditions and disease cycle

Typical symptoms of FCR-infected plants show brown discoloration at the subcrown internode region. Depending upon the disease severity, the plant may show early maturity and produce white heads with no or shrivelled grains (Kazan and Gardiner, 2018). A soil water potential of -0.3 to -0.7 MPa with at least 12°C conditions is required for disease development at the seedling stage (Alahmad et al., 2018). The fungi survive as mycelium or chlamydospores in crop residue. The soil-borne conidia or infected crop residue serve as the secondary infection for the disease (Kazan and Gardiner, 2018).

### 10.2 Cultural, biological and chemical control

The ability of the fungus to survive for five years in soil and become a chronic problem makes it necessary for growers to manage the crop stubble effectively to control the disease (Kazan and Gardiner, 2018; Jin et al., 2020). Cultural practices including crop rotation with non-host such as sorghum, chickpea, and lentil, use of high-quality treated seed, proper nitrogen fertilizer rates, and adjusting planting dates so that crop matures under dry conditions result in reducing the crop loss due to FCR. *Bacillus* and *Trichoderma harzianum* has been a great success in glasshouse study for effectively controlling the FCR (Moya-Elizondo and Jacobsen, 2016). Seed dressing or foliar spray of epoxiconazole and carbendazim can control the FCR (Kazan and Gardiner, 2018).

### 10.3 Breeding for fusarium crown rot disease resistance

The unavailability of complete resistance in current genotypes against FCR creates many opportunities for plant breeders. Most studies have been conducted under controlled conditions for detecting QTL conferring resistance to FCR (Jin et al., 2020). More field experiments are required to confirm the source of FCR resistance. However, the list of QTLs associated with FCR resistance is compiled in **Table 2**.

## 11 Take-all

Take-all is a common worldwide root disease of wheat caused by the soil-borne fungus *Gaeumannomyces graminis* var *tritici* (Hernández-Restrepo et al., 2016; Gargouri et al., 2020). The pathogen has a wide range within the *Poaceae* family, of which wheat (*Triticum aestivum*), barley (*Hordeum vulgare*), and oats (*Avena sativa*) are the major hosts. The ‘Take-all’ was originally named by the farmers of South Australia, where it completely destroyed wheat fields (Cook, 2003). Take-all decline (TAD) is a natural phenomenon by which microorganism activity, specifically the 2,4-DAPG producing *Pseudomonas* species, reduces the severity and incidence of take-all disease. This phenomenon happens after the severe outbreak of take-all disease on a continuous wheat or barley cropping system (Cook, 2003; Kwak and Weller, 2013). The take-all disease tends to persist only on cropland that practices continuous wheat or barley without considering proper crop rotation practices (Cunfer et al., 2006).

### 11.1 Symptoms, favorable conditions and disease cycle

Take-all infection begins with root rot and later spreads to the crown and interrupts the water transport into plants. The aerial symptoms include stunted plants, premature ripening, and whiteheads which can be confused with other soil-borne diseases like fusarium foot rot (Cook, 2003; Kwak and Weller, 2013; Gargouri et al., 2020). The disease can develop at a soil pH of 5.5 to 8.5, and the optimum temperature for its growth is 20-25°C. The pathogen highly favors light-textured soils having alkaline pH. Moisture in the soil helps the pathogen to spread from one place to another. It survives saprophytically as mycelium on the residue of infected crops. The primary infection is caused by mycelium, which later causes secondary infection and gets transmitted *via* contact with the susceptible roots (Bailey and Gilligan, 1999).

### 11.2 Cultural, biological and chemical control

Practices including crop rotation with non-host crops, including canola (*Brassica napus*), applying a balanced rate of ammonium fertilizer, and delayed seeding help control the disease. Seed inoculation with specific *Pseudomonas* spp. also helps in reducing the incidence of take-all disease in wheat (Cook, 2003). Furthermore, seed treatment with a recommended fungicide containing triadimenol (Baytan^®^) reduces the disease occurrence (Cook, 2003).

### 11.3 Breeding for take-all resistance

Identification and development of a tolerant cultivar against take-all disease are required. No effective resistance against take-all has been found in available wheat cultivars (Zhang et al., 2019b). Breeding efforts for introgression resistance from wild wheat relatives have been proven valuable in the past. Recently, researchers have identified *H139*, a 2Ns/2D substitution line which showed improved resistance against take-all (Bai et al., 2020). However, there is no information available on different genes/QTLs that have been identified for imparting resistance against take-all disease in wheat (Liu et al., 2013; Bai et al., 2020; Sheng-sheng et al., 2020).

## 12 Bacterial leaf streak

Bacterial leaf streak (BLS) also called as black chaff is an important bacterial disease of small grain crops, especially wheat. This disease was first reported on barley (Jones et al., 1916) and later on wheat (Smith et al., 1919). It is caused by a gram-negative, non-spore forming, rod shaped bacterium: *Xanthomonas translucens* pv. *undulosa* (*Xtu*). *Xtu* releases type- III effectors into plants to modulate virulence (White et al., 2009). BLS is sporadic in nature (Ramakrishnan et al., 2019).

BLS has worldwide distribution reported in North America, South America, Australia, Africa, Asia, Near and Middle East. In the USA, this disease is more prevalent in lower mid-south regions however, recently more reports are coming out about increasing prevalence of this disease in the upper Midwest of of the United States (Adhikari et al., 2012).

Damage ranges from negligible to as high as 40% and is dependent on the stage of infection and disease pressure (Shane et al., 1987; Forster and Schaad, 1988). BLS also affects the protein content hence, deteriorating the grain quality (Shane et al., 1987). Bacterium has a wide host range among cereals and infects wheat, triticale, barley, rye, oats, and rice (Adhikari et al., 2011). *Xtu* was also reported to be infecting asparagus which is suggesting an expansion of host range for this pathogen (Rademaker et al., 2006).

### 12.1 Symptoms, favorable conditions and disease cycle

Infection can occur on leaves, peduncles, spikes, and rachis. On leaves, development of translucent water soaking streaks along and between leaf veins are the initial symptoms. Further, these streaks coalesce and form large brown necrotic areas. Under humid weather, milky bacterial exudates can be seen from these streaks. On spikes, symptoms appear as dark purple to black lesions on the glume, giving disease the name “Black Chaff”. Under field conditions, differentiation for black chaff and stagonospora nodorum blotch is difficult.

BLS is more prevalent in warmer and high rainfall areas (Duveiller et al., 1997). There are mainly three factors: rainfall, temperature, and wind speed to be significantly related to development of BLS. Sprinkler irrigation can increase the chances of occurrence of this disease. Bacteria tolerates a wide range of temperatures ranging from 15-30°C. Lower temperatures retards pathogen multiplication and disease spread. Symptoms development occurs when the bacterial population reaches a level of 108cfu/leaf (Duveiller et al., 1992). *Xtu* also have ice-nucleating activity by which they trigger ice formation and cause damage to plant tissue, making them suitable for invasion and multiplication (Sands and Fourrest, 1989).

Seeds are the primary source of inoculum of *Xtu* (Jones et al., 1916; Smith et al., 1919). *Xtu* can survive on seeds for 63-81 months depending upon the storage conditions (Forster and Schaad, 1988). *Xtu* can also survive in soil and crop residue. More than 1,000 colony forming units (cfu) per gram of seed are required for the BLS epidemic to occur. Pathogens can also survive on grasses like canary grass (*Phalaris canariensis* L.), brome (*Bromus inermis* Leyss.), and timothy (*Phleum pratense* L.) (Duveiller et al., 1992).

Bacteria enter into interior parts of the leaf through stomata or wounds, multiply in parenchyma in large masses and produce elongated streaks over the leaf surface. Later on, milky/yellow exudates from leaf surface releases the bacterium and is disseminated to the whole field by wind or rain (Cunfer, 1987; Duveiller et al., 1992).

### 12.2 Cultural and chemical control

Using certified seeds or treating seeds with bactericide (copper containing compounds such as Kocide, Cuprofix Ultra and Champ) can be helpful in reducing the inoculum load. Alternating with non-host crops and removal of grasses may help in managing the disease (Duveiller et al., 1992). Application of Silicon to soil can also help in achieving resistance against BLS (Silva et al., 2010). However more research needs to be done over this approach.

However, treating seeds with cupric acetate (0.5%) at 45°C for 20 minutes can reduce the seeds-borne infection (Duveiller et al., 1992). Seed treatment with guazatine (300g/L) and imazalil (20gm/L), formalin and dry heat may also reduce the infection. Foliar sprays with mono- and di-potassium salts of phosphorus may be helpful in managing the disease (Rodríguez-García et al., 2020).

### 12.3 Breeding for bacterial leaf streak disease resistance

A large number of wheat germplasm have been evaluated till date against BLS resistance, however no immune or high-level resistance cultivars have been found yet. Only partial resistance is known yet as resistance is quantitatively controlled (Duveiller et al., 1992). During early 1990’s, researchers found five genes (*Bls1*, *Bls2*, *Bls3*, *Bls4*, *Bls5*) in wheat imparting partial BLS resistance (Duveiller et al., 1992). Recently, a major gene *Xct1* imparting resistance against BLS in triticale was recorded which can be transferred to wheat (Wen et al., 2018). QTLs associated with BLS are compiled in **Table 1**.

## 13 Barley yellow dwarf virus

Barley Yellow Dwarf (BYD) is the most serious, widespread, economically damaging viral disease of small grains including wheat, barley, and oats worldwide. It is caused by a group of phloem-restricted viruses belonging to the family *Luteoviridae* and are transmitted by aphids. Barley yellow dwarf virus PAV (BYDV-PAV) is the dominant serotype causing this harmful disease (Griesbach et al., 1990) and is transmitted by *Rhopalosiphum padi* (bird cherry-oat aphid) and *Sitobion avenae* (English grain aphid) (Kaddachi et al., 2014). BYDV is likely to have originated in the grasses native to North America and was first recognized as a problem in the USA in 1890 in oats and was widespread in the midwest (Walls et al., 2019). Estimates made are that with each 1% increase in BYDV incidence, yield losses for wheat can be 13-25 kg/ha and can extend up to 27-45 kg/ha (McKirdy et al., 2002). Average yield losses vary from 11 to 33% whereas losses can go as high as 80% (McKirdy et al., 2002; Kaddachi et al., 2014). BYDVs are of global importance and infect most grasses but are more damaging in oats, barley, and wheat (Miller and Rasochová, 1997).

### 13.1 Symptoms, favorable conditions and disease cycle

Symptom development varies depending upon genotype, environmental conditions, virus strain and time of infection (Kaddachi et al., 2014). Symptoms start appearing approximately after 14 days of infection. Symptoms can be confused with wheat streak mosaic virus, root or crown disease, nutrient deficiency or any other environmental stress (https://cropwatch.unl.edu/plantdisease/wheat/barley-yellow-dwarf). The most obvious symptoms of BYDV in wheat are chlorosis of leaf blades (especially tips) alongside vascular bundles, stunting of plants and reduced tillering (Miller and Rasochová, 1997; Choudhury et al., 2018). Virus also interferes with plant physiological processes like photosynthetic gas exchange, chlorophyll content, chlorophyll fluorescence and affected plants are more vulnerable to biotic and abiotic stresses (Choudhury et al., 2019a). Infection by BYDV causes reduction of photosynthesis per plant (Choudhury et al., 2019b). Most effective migration of aphids carrying BYDV occurs in cool and moist season temperatures varying from 10°-15°C. Symptom expression is favored by bright and sunny weather and appears after 2 weeks of infection (https://cropwatch.unl.edu/plantdisease/wheat/barley-yellow-dwarf). Aphids acquire the virus from infected phloem cells and aphids remain viruliferous for the rest of their life. Aphids carrying BYDV transmit viruses to healthy plants as they feed on them and the cycle continues (Walls et al., 2016).

### 13.2 Cultural control

Altering planting dates to a time when aphids activity is less, is helpful in managing this disease (Bockus et al., 2016; Walls et al., 2016). Controlling grassy weeds and volunteer cereals like maize can help in managing this disease (Rashidi et al., 2020).

### 13.3 Breeding for barley yellow dwarf virus resistance

Both tolerance and resistance mechanisms can occur. *Bdv1* was identified as the dominant gene located on the short arm of chromosome 7D in wheat imparting tolerance against BYDV. *Bdv1* was found to be linked with leaf and stripe rust resistance genes (Lr34 and Yr18) (Singh et al., 1993). *Ryd1*, *Ryd2*, *Ryd3* and *RYd4Hb* were the genes identified in barley imparting tolerance (Niks et al., 2004; Scholz et al., 2009).

Wheat lacks natural resistance against BYDV. However, wild relatives of wheat most importantly *Thinopyrum* spp. harbor some important BYDV resistance genes, few of which have been transferred to commercially wheat varieties *via* developing translocation lines (Sharma et al., 1995; Ayala et al., 2001). Breeding programmes exploit these wild relatives to find novel sources of resistance against BVDV. *Bdv2*, the first and most-widely used BYD resistance gene in breeding programmes was a result of 7D-7Ai# translocation (Hohmann et al., 2011). Similarly, *Bdv3* was the result of 7B-7Ai# translocation (Kong et al., 2009) and *Bdv4* of 2D-2Ai# translocation (Lin et al., 2006). These resistance genes have diverse mechanisms of action and works by either interfering with cell-to-cell movement or disrupting viral replication (Sharma et al., 1989; Anderson et al., 1998). QTLs associated with BYDV are compiled in **Table 1**.

## 14 Powdery mildew

Powdery mildew (PM) is one of the most destructive foliar diseases of wheat that is ranked as the eight most yield reducing pest at the global scale (Savary et al., 2019). The infection of powdery mildew can prevail throughout the year at different wheat producing areas around world. Wheat infected with powdery mildew suffers from both qualitative (grain end-use quality) and quantitative (final grain yield) losses (Alemu et al., 2021). Crops infected with powdery mildew results in decreased chlorophyll content and reduced photosynthetic activity (Feng et al., 2017). It is observed that under the severe disease infection, crop production losses can go up to 60%, though it usually causes a yield reduction of 5-40% (Draz et al., 2019). Earliest documentation on powdery mildew was demonstrated by Mains in 1933 and importance of powdery mildew was not realized until early 19^th^ before the use of increased rate of fertilizer use (Hermansen, 1968).

Powdery mildew is caused by an obligate biotrophic fungus, *Blumeria graminis* f. sp. *tritici* (*Bgt*) (Savary et al., 2019; Li et al., 2019). It was indicated in the global genomic analyses that wheat powdery mildew originated in the Fertile Crescent, and from there it spread across all Eurasia and eventually to other continents resulting from active human migration and trade (Sotiropoulos et al., 2022). It is particularly important for the south eastern region of the United States (Cowger et al., 2012), however, with the advancement in agronomic practices with increased plant population per unit area and fertilizer inputs, this disease is observed in some of warmer-drier regions of the world as well (Dubin and Duveiller, 2011; Lackermann et al., 2011; Cowger et al., 2012).

### 14.1 Symptoms, favorable conditions and disease cycle

The symptoms of powdery mildew can be detected on any plant part, the distinguishing symptoms of powdery mildew include the presence of whitish powdery mycelia on whole leave area, eventually reducing the leaf area for photosynthesis (Mehta, 2014). As the disease grow on plant, black colored cleistothecia bodies interlaced within the whitish mycelia can be observed. Infected crop produce shriveled grain and early infection can results in reduced emergence (Mehta, 2014).

Pathogen causing powdery mildew produces two types of infectious spores i.e., conidia and ascospores. These spores germinate under humid environment conditions and later require 10–22°C coupled with dry environment for proper disease development (Te Beest et al., 2008; Kang et al., 2020). Upon successful disease establishment, a specialized germ tube is produced and elongates to form a thread-like hypha and later develop haustorium to withraw nutrients from the infected plant part (Acevedo-Garcia et al., 2017). Fungus mainly overwinters on wheat straw as chasmothecia.The primary spread of infectious powdery mildew spores to long distances is mainly accomplished *via* wind, but, the presence of heavy rains washes away the spores and eventually reduces the disease incidence (Mehta, 2014).

### 14.2 Cultural and chemical control

In spite of the use of improved cultivars, incidence of powdery mildew infection is still a major concern in the wheat growing areas especially if the infection occurs during seedling stage (Simeone et al., 2020). Crop rotation with non-host crops is one of the important cultural practices that can help reducing the buildup of pathogen inoculum in soil. Other practices including the use of high rate of nitrogen fertilizer, increased seed rate, limited plant spacing should be avoided (Jarvis et al., 2002; Janvier et al., 2007; Mehta, 2014). For effective chemical control, fungicide application is encouraged to be applied as seed dressing. Sulfur was the first fungicide used to control powdery mildew, later benzimidazoles, and triazoles were also suggested. The application of fungicide should be done at the emergence of flag leaf before boot starts appearing (Leath and Bowen, 1989; Golzar et al., 2016).

### 14.3 Breeding for powdery mildew resistance

A number of genes imparting resistance to powdery mildew have been documented and characterized over the last 30 years (Kang et al., 2020). Over 60 loci for Pm resistance have been described in wheat and its relatives (Hewitt et al., 2021). To date, 12 PM resistance genes have been cloned and are listed in **Table 2** (Wu et al., 2022). Most cloned R genes are NBS-LRR type, and the rest are kinases. These genes provide either race-specific resistance (qualitative resistance) or broad-spectrum resistance (quantitative resistance). Quantitative resistance is more durable and robust than qualitative resistance. Multiple studies reported the loss of R-gene-mediated resistance because of pathogen evolution, say Pm8 in China, Pm3a, Pm4a, Pm 17 in the USA, and Pm4b, Pm7, Pm24, Pm28 in Australia. This requires further screening of diverse germplasm or gene stacking (Kang et al., 2020). Susceptibility genes have also been shown to play a role in developing durable resistance. A well-studied S gene is an example of the Mlo gene locus and has been known to impart resistance against PM over more than 40 years (Várallyay et al., 2012). Enhanced disease resistance 1 (EDR1) also has been used in improving wheat resistance against powdery mildew (Zhang et al., 2017).

**Table 2:** List of QTLs and their positions on chromosome conferring resistance to different diseases of wheat.

Disease	Pathogen	Wheat Type	Mapping population	No. of QTLs/MTA	Chromosome	R^2^	Reference
Snow Mold	*T. ishikariensis and F. nivale*	Winter wheat	Biparental mapping	2	5D and 6B	12 to 14%	(Nishio et al., 2020)
Snow Mold	*T. ishikariensis and F. nivale*	Winter wheat	Biparental mapping	6	1A, 3A, 3B, 3D, and 6B	0.06-0.1%	(Kruse et al., 2019)
Snow Mold	*T. ishikariensis and F. nivale*	Winter wheat	Association mapping	100	17 out of 21 chromosomes	0.01 to 11.6%	(Lozada et al., 2019)
Snow Mold	*T. ishikariensis*	Winter wheat	Biparental mapping	2	5A and 6B	8-10%	(Kruse et al., 2017)
Eyespot	*O. yallundae*	Winter wheat	Association mapping	73	9 out of 21 chromosomes	0.03-0.06%	(Lewien et al., 2018)
Eyespot	*O. acuformis*	Winter and Spring wheat	Association mapping	108	9 out of 21 chromosomes	5-6%	(Zanke et al., 2017)
Eyespot	*O. acuformis and O. yallundae*	Spring wheat	Biparental mapping	1	5A	23 to 34%	(Burt et al., 2011)
Cephalosporium Stripe	*C. gramineum*	Winter wheat	Biparental and association mapping	80	11 out of 21 chromosomes	0.06-0.12%	(Froese et al., 2016)
Cephalosporium Stripe	*C. gramineum*	Winter wheat	Biparental mapping	15	3B, 4B, and 5A	4.2-18.0%	(Vazquez et al., 2015)
Cephalosporium Stripe	*C. gramineum*	Winter wheat	Biparental mapping	7	2B, 2D, 4B, 5A, and 5B	4.9-12.4%	(Quincke et al., 2011)
Common Root Rot	*B.sorokiniana*	Spring wheat	Biparental mapping	3	1 BS, 3BS, and 5BS	8.5-17.6%	(Zhu et al., 2014)
Common Root Rot	*B.sorokiniana.*	Spring wheat	Biparental mapping	4	2AL, 2BS, 5BL, and 6DL	63.1%	(Kumar et al., 2009)
Common Root Rot	*B.sorokiniana*	Spring wheat	Association mapping	4	1A, 3B, 7B, and 7D	1.4-2.6%	(Adhikari et al., 2012)
Common Root Rot	*B.sorokiniana*	Spring wheat	Association mapping	1	7B	14%	(Jamil et al., 2018)
Common And Dwarf Bunt	*Tilletia caries and T. controversa*	Winter wheat	Association mapping	123	14 out of 21 chromosomes	1-9%	(Mourad et al., 2018)
Common And Dwarf Bunt	*Tilletia caries and T. controversa*	Winter wheat	Biparental mapping	2	6D and 7A	35%	(Wang et al., 2019)
Common And Dwarf Bunt	*Tilletia caries and T. controversa*	Winter wheat	Biparental mapping	3	1A, 2B, and 7D	32-56%	(Chen et al., 2016)
Karnal Bunt	*T. indica*	Spring wheat	Biparental mapping	2	5B and 6B	32%	(Singh et al., 2007)
Karnal Bunt	*T. indica*	Spring wheat	Biparental mapping	3	2A, 4B and 7B	25%	(Sukhwinder-Singh et al., 2003)
Fusarium Crown Rot	*F. Pseudograminearum*	–	Biparental mapping	3	6A, 2D, amd 2A	5.24-10.17%	(Yang et al., 2019)
Fusarium Crown Rot	*F. Pseudograminearum*	–	Association mapping	2	5DS and 2DL	20.2-31.1%	(Zheng et al., 2014)
Fusarium Crown Rot	*F. Pseudograminearum*	–	Association mapping	1	1DL	17.4%	(Martin et al., 2015)
Bacterial Leaf Streak	*X. translucens pv. undulosa*	Spring Wheat	Association mapping	5	1A, 4A, 4B, 6A, 7D	1.4 to 2.6%	(Adhikari et al., 2012)
Bacterial Leaf Streak	*X. translucens pv. undulosa*	Spring Wheat	Association mapping	4	1A, 5A, 5D, 6B	14.3%	(Gurung et al., 2014)
Bacterial Leaf Streak	*X. translucens pv. undulosa*	Spring Wheat	Identity by descent mapping	2	2A, 6B	0.5 to 29.5%	(Kandel et al., 2015)
Bacterial Leaf Streak	*X. translucens pv. undulosa*	Winter Wheat	Association mapping	5	1AL, 1BS, 3AL, 4AL, 7AS	42%	(Ramakrishnan et al., 2019)
Barley Yellow Dwarf Virus	BYDV-PAV	Spring Wheat	Bi-parental mapping	22	12 out of 21	3.7-15.8%	(Ayala et al., 2002)
Barley Yellow Dwarf Virus	BYDV-PAV	Spring Wheat	Bi-parental mapping	7	5 out of 21	4.1-13.3%	(Ayala et al., 2002)
Barley Yellow Dwarf Virus	BYDV-PAV	–	Association mapping	4	2A, 2B, 6A, 7A	0.23%	(Choudhury et al., 2019c)
Barley Yellow Dwarf Virus	BYDV-PAV	Winter Wheat	Bi-parental mapping	3	5A, 6A , 7A	7.1-25%	(Choudhury et al., 2019c)

## 15 Wheat streak mosaic virus

Wheat streak mosaic (WSM), caused by Wheat Streak Mosaic Virus (WSMV), is a common disease in many wheat-growing regions in the U.S. and the world. WSMV is transmitted by the wheat curl mite. WSMV is a single-stranded, monopartite, positive sense RNA virus and type member of the genus *Tritimovirus* in the family *Potyviridae*. WSM was first observed in Nebraska by Peltier in 1922. WSMV is hosted by many plant species of family *Poaceae*. Because of the devastating impact of the diseases, it has been a concern because losses can range from minimal to complete crop failure.

### 15.1 Symptoms, favorable conditions and disease cycle

Symptoms start on young leaves as pale green streaks which elongate to form yellow stripes, forming a mosaic pattern that runs parallel to leaf veins. These symptoms are easily confused with nutritional disorders, chemical damage, or environmental effects. In a severe epidemic, plants in the whole field become symptomatic (Singh et al., 2018). In winter wheat, serious infection occurs when the disease starts in the autumn. The appearance of symptoms in autumn gives an indication of severe epidemics in following spring. Infected plants appear stunted, less upright, and yellow and poorly tillered in spring. Yellowing intensifies with an increase in temperature. Severely infected plants may not have spikes or are poorly filled with shriveled kernels. The effects of spring infections on symptom development and yield are usually subtle (Tatineni and Hein, 2018).

In winter wheat, initial infection occurs during autumn when mites move from other cereal hosts or volunteer wheat to newly emerged wheat, and during this step they transmit WSMV. The amount of damage caused is determined by the density of mite population, prevailing temperature, virus host’s proximity to the wheat field during planting, cultivar susceptibility, and time of infection (Tatineni and Hein, 2018). WSMV overwinters in live tissue of wheat plants, while mites overwinter as eggs, larvae, nymphs, and adults in the crown. In spring, with the temperature rise, mites become active and spread in different fields. At the heading, mites move from above-ground parts to infect the spikes. After wheat maturity, these mites find new hosts with green tissue to feed and survive the remaining summer. Following planting in the autumn, the mites move onto the newly emerged wheat and transmit WSMV, completing the disease cycle (Singh et al., 2018).

### 15.2 Cultural and chemical control

As WMCVs cannot be controlled by chemicals and chemical control for vectors is infective, hence, most effective strategy is to use cultural practices. Management of over-summering hosts for controlling mites is the main cultural control for controlling the disease development in winter. Delayed planting can also avoid the virus infections, however that comes with consequence of wheat yield due to fewer growing days before the onset of winter. Pre-harvest volunteer wheat should be controlled with proper herbicides and tillage. To be effective, volunteer wheat should be completely dead at least two weeks prior to planting.

### 15.3 Breeding for wheat streak mosaic virus

Minimal success has been achieved for resistance to WSMV, however, efforts to transfer resistance genes from closed relatives have generated some success. So far, two resistance genes (Wsm1 and Wsm3) have been transferred by chromosomal translocation from *Thinopyrum intermedium* (Friebe et al., 1991). However, these two translocations have resulted in minimum success due to linkage drag for the breeding programs. A third gene was identified, with no clear origin and it has now been incorporated into several breeding lines. Furthermore, WCM virus resistant varieties can aid in management of WCMVs. Four curl mite colonization (*Cmc*) genes have been identified and are being used in breeding programs. Resistant genes have been transferred from *Secale cereal* and *Agropyron elongatum* to impart resistance.

## 16 Future perspectives

Exponential increase in cloning for resistance genes in wheat is expected due to sequencing of the Chinese spring wheat genome, the hexaploid wheat reference genome which came out in 2018 (https://www.wheatgenome.org). Recently, a collaborative effort from ten countries published reference quality sequences and scaffold level assemblies of 10 and 5 popular hexaploid wheat varieties respectively (Walkowiak et al., 2020). This resulted in expansion of genome assembly available for researchers to work with. Wild relatives are resistant to multiple diseases which will aid in utilizing those relatives to transfer resistance to elite and commercially grown cultivars.

Better comprehension of plant-pathogens interactions especially for hemi-biotrophic and necrotrophic pathogens; finer understanding of pattern triggered immunity and effector triggered immunity especially hormonal changes will help in achieving more in-built resistance. Manipulation of susceptibility genes has a capability in changing the way the resistance against diseases is strived for. Most of the wheat cultivars are not amenable to transformation, there is a likely scope of research in this field which will ultimately help in fast cloning of genes.

Development of reliable weather prediction models will allow growers to alter their spray dates depending on the disease intensity at that period of year. There is a huge potential in utilizing high throughput screening methods such as imaging and remote sensing to monitor different crop growth stages. GWAS was unable to aid in selection of disease resistant lines, genomic selection can be employed to select for disease resistant lines. There is a need for collaborative efforts by breeders, pathologists, geneticists, biologists, and biochemists to achieve complete resistance against diseases.

## Author contributions

JS, BC, and KS: Outline the study and wrote the manuscript. JS, BC, AR, SY, and KS: reviewed and made the submission ready document. and SHY: provided the funds for the project. All authors contributed to the article and approved the submitted version.
